# Impact of Bevacizumab on parenchymal damage and functional recovery of the liver in patients with colorectal liver metastases

**DOI:** 10.1186/s12885-016-2095-6

**Published:** 2016-02-10

**Authors:** Andreas M. Volk, Johannes Fritzmann, Christoph Reissfelder, Georg F. Weber, Jürgen Weitz, Nuh N. Rahbari

**Affiliations:** Department of Visceral, Thoracic and Vascular Surgery, University Hospital Carl Gustav Carus, Technical University Dresden, Fetscherstr. 74, D-01307 Dresden, Germany

**Keywords:** Bevacizumab, Chemotherapy, Liver resection, Parenchymal damage, Complications

## Abstract

**Background:**

Little is known about the safety of the anti-VEGF antibody bevacizumab in patients undergoing resection for colorectal liver metastases (CLM). This meta-analysis evaluates the impact of bevacizumab on parenchymal damage and functional recovery in patients undergoing resection for CLM.

**Methods:**

The Medline, Embase and Cochrane Library were systematically searched for studies on preoperative chemotherapy with and without bevacizumab prior to resection of CLM. Studies that reported histological and/or clinical outcomes were eligible for inclusion. Meta-analyses were performed using a random effects model.

**Results:**

A total of 18 studies with a total sample size of 2430 patients (1050 patients with bevacizumab) were found. Meta-analyses showed a significant reduction in sinusoidal obstruction syndrome (SOS) (Odds ratio 0.50 [95 % confidence interval 0.37, 0.67]; *p* < 0.001; I^2^ = 0 %) and hepatic fibrosis (0.61 [0.4, 0.86]; *p* = 0.004; I^2^ = 7 %) after preoperative chemotherapy with bevacizumab. The reduced incidence of posthepatectomy liver failure in patients with bevacizumab treatment just failed to reach statistical significance (0.61 [0.34, 1.07]; *p* = 0.08 I^2^ = 6 %). While there was no difference in perioperative morbidity and mortality, the incidence of wound complications was significantly increased in patients who received bevacizumab (1.81 [1.12, 2.91]; *p* = 0.02 I^2^ = 4 %).

**Conclusions:**

The combination of bevacizumab with cytotoxic chemotherapy is safe but increases the incidence of wound complications after resection of CLM. The reduction of SOS and hepatic fibrosis warrant further investigation and may explain the inverse association of bevacizumab administration and posthepatectomy liver failure.

**Electronic supplementary material:**

The online version of this article (doi:10.1186/s12885-016-2095-6) contains supplementary material, which is available to authorized users.

## Background

Complete surgical resection remains the only curative option in patients with colorectal liver metastases (CLM) enabling 5-year overall survival rates of 50 % [1, 2]. Effective oxaliplatin- and irinotecan-based chemotherapy protocols together with targeted agents have significantly improved objective response rates, conversion to resectability and long-term survival in metastatic colorectal cancer not amenable to curative resection [3–6]. As a consequence of the increased use of modern combination chemotherapy protocols, a growing number of patients undergo hepatic resection after treatment with cytotoxic and molecular targeted agents. Hepatic toxicity of irinotecan and oxaliplatin-containing regimens are well-described and typically manifest as chemotherapy-associated steatohepatitis (CASH) and sinusoidal obstruction syndrome (SOS), respectively. However, much less is known about the effects of targeted agents on parenchymal damage to the liver and their influence on perioperative outcome after hepatic resection. Among targeted agents approved for treatment of metastatic colorectal cancer, the impact of bevacizumab, a monoclonal antibody against the vascular endothelial growth factor A (VEGF-A) on liver histology and perioperative complications is of particular interest. Besides its role in pathological angiogenesis, the VEGF family of growth factors exerts important physiological functions. The important function of VEGF in homeostasis of the liver microenvironment, liver regeneration and wound healing have therefore raised concerns about the safety of bevacizumab in the peri-operative setting of patients undergoing hepatic resection. To date, several reports have been published on the effects of bevacizumab on liver-parenchymal damage, functional recovery and perioperative outcome after resection of CLM with in part conflicting results [7–10].

The aim of this systematic review and meta-analysis was to evaluate the effects of preoperative bevacizumab administration on histological and perioperative outcomes of patients undergoing surgical resection of CLM.

## Methods

This systematic review and meta-analysis was conducted in accordance to the PRISMA statement [11].

### Search strategy and selection criteria

A computerized search of the Medline, Embase and Cochrane Library databases was performed in May 2014 using the following search terms in various combinations: ‘Colon’, ‘Rectal’, ‘Colorectal’, ‘Liver’, ‘Hepatic’, ‘Metastases’, ‘Bevacizumab’, ‘Avastin’. To find other potentially eligible studies, the reference lists of relevant articles were searched manually. First, the search findings were screened for potentially eligible studies based on the titles and abstracts. For references that were considered potentially relevant, the full articles were obtained for detailed evaluation using the following selection criteria: All studies (prospective or retrospective) that reported the impact of preoperative bevacizumab administration on perioperative outcome and/or liver histology of patients undergoing resection of CLM were eligible for inclusion. For studies to be eligible for inclusion, at least one predefined outcome for patients treated with chemotherapy with and without addition of bevacizumab had to be reported within one study/report. Comments and letters were excluded as were studies that were not published in a peer-reviewed journal. Furthermore, studies that were published in a language other than English, German or French were excluded. In case of multiple publications from the same institution with identical or overlapping patient cohorts the most informative report was included.

### Data extraction and quality assessment

Two authors (N.N.R. and A.M.V.) independently extracted the following data from each identified study: first author, year of publication, study period, study, design, sample size, baseline characteristics of the study cohort, kind of concomitant chemotherapy, number of preoperative chemotherapy cycles with Bevacizumab, time interval between last Bevacizumab administration and surgery. The following histological parameters were recorded separately for patients with and without preoperative administration of Bevacizumab: sinusoidal obstruction syndrome (total and moderate/severe), hepatic fibrosis, hepatic steatosis, complete pathological response and complete (R0) tumor resection. With regard to perioperative outcomes data on the following endpoints were documented: perioperative morbidity and mortality, wound complications, liver failure. Disagreements were resolved by discussion.

To assess the methodological quality of included studies, the risk of bias tool recommended by the Cochrane Collaboration was applied [12]. The criteria proposed by the Grading of Recommendations, Assessment, Development and Evaluation (GRADE) Working Group (www.gradeworkinggroup.org) were used for evaluation of non-randomized studies [13–15]. The following criteria were evaluated for each included study: application of adequate eligibility criteria, adequate measurement of outcomes, adequate control of confounding factors, completeness of follow-up and adequacy of its duration, adequate reporting of outcomes and absence of other sources of bias. The use of scales with scores for multiple items that are summed up is discouraged by the Cochrane Collaboration. The above criteria were therefore used to grade individual studies as high or low risk of bias [12, 15, 16].

### Statistical analyses

Meta-analyses were performed for outcomes for which at least two of the included studies provided comparative data for patients who underwent liver resection after preoperative chemotherapy with and without Bevacizumab. Odds ratio (OR) was chosen as effect measure dichotomous data, which was reported together with the 95 % confidence interval (CI). Meta-analyses were carried out using a random effects model for more conservative effect estimates due to potential inter-study heterogeneity regarding study populations, chemotherapy protocols and definitions of outcome parameters [17]. Heterogeneity was assessed with I^2^ statistics. This approach describes the proportion of total variation observed between the trials that is attributable to differences between trials rather than sampling error (chance) [18]. Moderate to high degree of statistical heterogeneity was assumed in case of an I^2^ value of more than 30 %. Reasons for statistical heterogeneity were explored using sensitivity analyses (exclusion of individual studies). Furthermore, subgroup analyses carried out to evaluate the impact treatment duration, time interval between last bevacizumab treatment and surgery and kind of concomitant chemotherapy on the results. Presence of publication bias was evaluated using Funnel plot analyses [19].

Meta-analyses were carried out using Review Manager Version 5.0 software (Copenhagen: The Nordic Cochrane Centre; The Cochrane Collaboration, 2008).

## Results

**Fig. 1:**
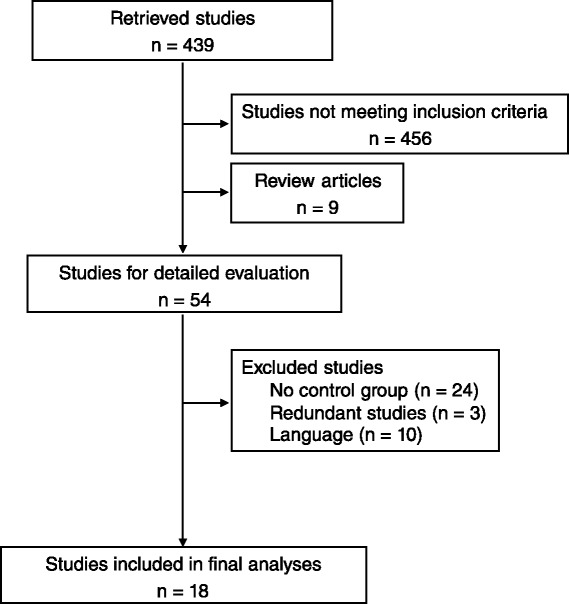
Flow chart of study selection

**Fig. 2:**
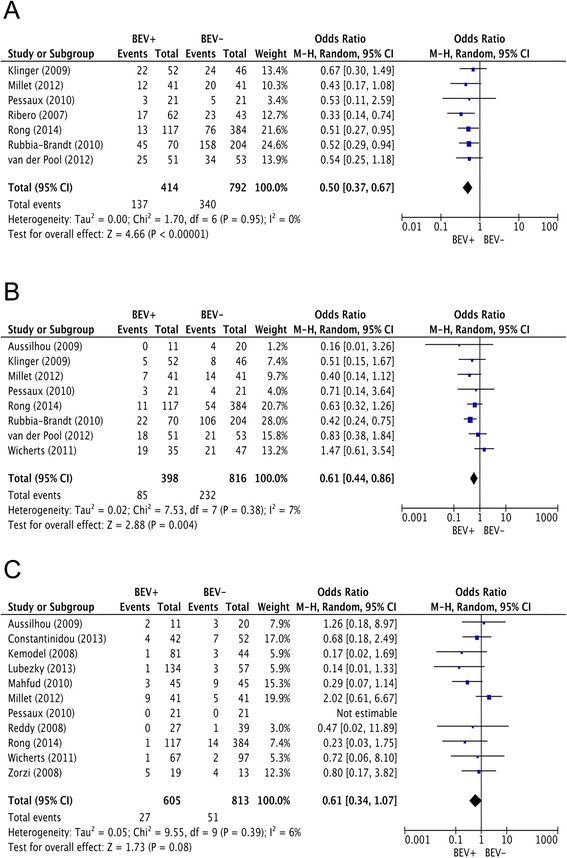
Meta-analyses on the association of preoperative bevacizumab treatment with parenchymal damage and posthepatectomy liver failure in patients with CLM. **a** Association of bevacizumab treatment with sinusoidal obstruction syndrome (SOS). **b** Association of bevacizumab treatment with hepatic fibrosis. **c** Association of bevacizumab treatment with posthepatectomy liver failure

The systematic literature search identified 18 relevant studies (Fig. 1) [7, 8, 10, 20–34]. These studies had a cumulative sample size of 2430 patients, of which 1050 patients received bevacizumab prior to resection of CLM (Table 1). The included studies were published between 2007 and 2014. More than six cycles of preoperative treatment with bevacizumab was administered in five studies [7, 20, 22, 26, 33], whereas in the remaining studies six or less cycles of chemotherapy with bevacizumab was given. The average time interval between the last dose of bevacizumab and the date of surgery was eight weeks or less in seven studies (Fig.2) [8, 22, 24, 25, 30, 33, 34] and more than eight weeks in eight studies [7, 10, 20, 21, 23, 26, 27, 31]. Bevacizumab was combined with oxaliplatin-based chemotherapy regimen in the majority (>76 %) of study patients in eight of the included studies [8, 24, 27–31, 34].

**Table 1:** Characteristics of identified studies

Reference	Year	Inclusion period	Sample size (total/BEV)	Study type	CTX in BEV group OX/IRI/OX + IRI [%]	No BEV cycles	Interval last BEV cycle to surgery	Risk of bias
Aussilhou	2009	2002–2008	40/13	Prosp. CS	54/38	12 (2–36)	92 days	High
Constantinidou	2013	Until 9/2010	94/42	Retrosp. CS	64/33	4.5 (4–12)^a^	73 (44–141) days	High
D’Angelica	2007	2004–2005	64/32	Matched CCS	56/37	9 (4–15)	6.9 (3–15) weeks	High
Kesmodel	2008	2004–2006	125/81	Retrosp. CS	70/36	84 (14–513)^b^	58 (31–117) days	High
Klinger	2009	2001–2006	106/56	Retrosp. CS^e^	100/0	5	5 weeks	High
Lubezky	2013	2000–2007	191/134	Retrosp. CS	72/28	-	>6 weeks	High
Mahfud	2010	2005–2007	90/45	Matched CCS	24/71	9 (7–10)^a^	9 weeks (60, 47–73 days)	Low
Millet	2012	2006–2011	82/41	Matched CCS	15/85	6 (4–16)^a^	65 (39–90) days	Low
Pessaux	2010	2005–2007	42/21	Matched CCS	76/10/14	8.1 ± 4.7	11.7 ± 4.7 weeks	Low
Reddy	2008	1996–2006	96/39	Retrosp. CS	79/21	6 (3–8)^c^	10 (8–13) weeks	High
Ribero	2007	2002–2006	105/62	Retrosp. CS	100/0	6 (3–12)^a^	>6 weeks	High
Rong	2014	2002–2012	501/117	Retrosp. CS	100/0	6 ± 12^a^	-	High
Rubbia Brandt	2010	–	274/70^d^	Retrosp. CS	100/0	-	-	High
Tamandl	2009	2005–2009	214/102	Retrosp. CS	82/13	6 (1–20)	34 (17–99) days	High
van der Pool	2012	2003–2008	104/51	Retrosp. CS	100/0	4 (1–15)^a^	11 (5–38) weeks	High
Vera	2014	2005–2011	95/51	Retrosp. CS	45/53	6 (1–21)^a^	-	High
Wicherts	2011	2005–2009	164/67	Retrosp. CS	23/68/2	8.6 (1–34)	8 (3–19) weeks	Low
Zorzi	2008	1995–2007	43/26	Retrosp. CS	100/0	5 (3–20)^a^	7.9 (3–36) weeks	High

^a^Number of CTx cycles

^b^Duration of BEV treatment in days

^c^Duration of BEV treatment in months

^d^Irinotecan was added in 79 patients of the whole study cohort

^e^Combined retrospective analysis of two phase II trials. Continuous data are presented as median (range) or mean (standard deviation) based on the kind of data presented in the original publication

### Histological analyses

A total of seven studies with 1206 patients provided data on SOS (Additional file 1: Table S1) [8, 10, 24, 26, 28, 29, 31]. Meta-analysis of the results from these studies showed a statistically significant reduction in SOS for patients who received chemotherapy with bevacizumab with no statistical heterogeneity (0.50 [0.37, 0.67]; *p* < 0.001; I^2^ = 0 %). This association was confirmed for the development of moderate and severe SOS (0.31 [0.18, 0.53]; *p* < 0.001; I^2^ = 37 %), which was reported in seven studies [8, 10, 20, 24, 26, 29, 31]. Sensitivity analyses revealed that heterogeneity was caused by the study of Aussilhou et al. [20]. Exclusion of this study completely removed statistical heterogeneity (0.25 [0.17, 0.38]; *p* < 0.001; I^2^ = 0 %). Subgroup analyses confirmed the protective effect of preoperative chemotherapy combined with bevacizumab on total as well as moderate/severe SOS throughout all evaluated strata (Table 2).

**Table 2:** Meta-analyses on outcomes of parenchymal damage and perioperative outcomes after preoperative chemotherapy with and without bevacizumab for CLM

Histological analyses					
	Subgroup	SOS	Moderate/Severe SOS	Hepatic fibrosis	Hepatic steatosis
Total	-	0.50 [0.37, 0.67]	0.25 [0.17, 0.38];	0.61 [0.44, 0.86]	0.96 [0.63, 1.45]
		*p* < 0.001; I^2^ = 0 %	*p* < 0.001; I^2^ = 0 %	*p* =0.004; I^2^ = 7 %	*p* = 0.83; I^2^ = 30 %
No of BEV cycles	≤6	0.50 [0.34, 0.72]	0.21 [0.11, 0.41]	0.60 [0.38, 0.94]	0.94 [0.56, 1.59]
		*p* < 0.001; I^2^ = 0 %	*p* < 0.001; I^2^ = 0 %	*p* = 0.03; I^2^ = 0 %	*p* = 0.82; I^2^ = 53 %
	>6	0.46 [0.21, 1.01]	0.29 [0.10, 0.81]	0.43 [0.19, 1.0]	0.93 [0.34, 2.52]
		*p* = 0.05; I^2^ = 0 %	*p* = 0.02; I^2^ = 0 %^a^	*p* = 0.05; I^2^ = 0 %^b^	*p* = 0.89; I^2^ = 31 %
Time last BEV cycle to surgery	≤8 weeks	0.47 [0.23, 0.95]	0.21 [0.10, 0.47]	-	-
		*p* = 0.04; I^2^ = 32 %	*p* < 0.001; I^2^ = 0 %		
	>8 weeks	0.50 [0.28, 0.86]	0.26 [0.12, 0.56]	0.61 [0.34, 1.09]	0.63 [0.21, 1.84]
		*p* = 0.01; I^2^ = 0 %	*p* < 0.001; I^2^ = 0 %	*p* = 0.09; I^2^ = 0 %	*p* = 0.39; I^2^ = 45 %
Cytotoxic chemotherapy^4^	Oxaliplatin high	0.46 [0.21, 1.01]	0.29 [0.10, 0.81]	0.43 [0.19, 1.00]	0.93 [0.34, 2.52]
		*p* = 0.05; I^2^ = 0 %	*p* = 0.02; I^2^ = 0 %^a^	*p* = 0.05; I^2^ = 0 %^b^	*p* = 0.89; I^2^ = 31 %
	Oxaliplatin low	0.51 [0.37, 0.69]	0.25 [0.16, 0.38]	0.52 [0.37, 0.75]	0.94 [0.56, 1.59]
		*p* < 0.001; I^2^ = 0 %	*p* < 0.001; I^2^ = 0 %	*p* < 0.001; I^2^ = 0 %	*p* = 0.82; I^2^ = 53 %
Functional Recovery and Perioperative Outcome					
		Morbidity	Wound complications	Liver failure	Mortality
Total	-	1.10 [0.88, 1.37]	1.81 [1.12, 2.91]	0.61 [0.34, 1.07]	0.60 [0.20, 1.82]
		*p* = 0.39; I2 = 10 %	*p* = 0.02; I2 = 4 %	*p* = 0.08; I2 = 6 %	*p* = 0.37; I2 = 0 %
No of BEV cycles	≤6	0.99 [0.75, 1.30]	1.22 [0.61, 2.42]	0.49 [0.21, 1.14]	0.54 [0.10, 2.93]
		*p* = 0.95; I2 = 0 %	*p* = 0.58; I2 = 0 %^c^	*p* = 0.10; I2 = 0 %	*p* = 0.47; I2 = 0 %
	>6	1.51 [1.08, 2.13]	1.88 [0.89, 3.99]	0.85 [0.36, 2.00]	0.46 [0.09, 2.41]
		*p* = 0.02; I2 = 0 %	*p* = 0.10; I2 = 0 %	*p* = 0.71; I2 = 16 %	*p* = 0.36; I2 = 0 %
Time last BEV cycle to surgery	≤8 weeks	1.06 [0.70, 1.59]	1.59 [0.50, 5.11]	0.50 [0.16, 1.57]	1.16 [0.14, 9.37]
		*p* = 0.80; I2 = 33 %	*p* = 0.43; I2 = 0 %	*p* = 0.24; I2 = 0 %	*p* = 0.89; I2 = 0 %
	>8 weeks	1.27 [0.92, 1.74]	1.45 [0.84, 2.50]	0.70 [0.32, 1.51]	0.47 [0.13, 1.71]
		*p* = 0.15; I2 = 0 %	*p* = 0.19; I2 = 0 %	*p* = 0.36; I2 = 23 %	*p* = 0.25; I2 = 0 %
Cytotoxic chemotherapy^d^	Oxaliplatin high	1.19 [0.87, 1.63]	1.47 [0.87, 2.49]	0.61 [0.28, 1.30]	0.55 [0.14, 2.19]
		*p* = 0.28; I2 = 17 %	*p* = 0.15; I2 = 0 %	*p* = 0.20; I2 = 29 %	*p* = 0.40; I2 = 0 %
	Oxaliplatin low	1.01 [0.74, 1.38]	3.19 [0.82, 12.35]	0.50 [0.16, 1.59]	0.70 [0.11, 4.47]
		*p* = 0.95; I2 = 6 %	*p* = 0.09; I2 = 51 %	*p* = 0.24; I2 = 0 %	*p* = 0.71; I2 = 0 %

^a^Results of sensitivity analyses after exclusion of the study by Aussilhou et al. [20]

^b^Results of sensitivity analyses after exclusion of the study by Wicherts et al. [33]

^c^Results of sensitivity analyses after exclusion of the study by Rong et al. [28]

^d^The subgroup analysis on the kind of systemic chemotherapy administered was based on the fraction of patients who received oxaliplatin or irinotecan. Studies in the oxaliplatin high group had > 76 % of patients who received oxaliplatin, whereas studies in the oxaliplatin low had ≤ 76 % patients with oxaliplatin. This cut-off was chosen based on the average proportion of patients with oxaliplatin in each study

The definition of significant fibrosis applied in the identified studies is summarized in Additional file 2: Table S2. Meta-analysis showed a significant reduction of hepatic fibrosis in patients who received preoperative chemotherapy with bevacizumab before resection of CLM (0.61 [0.4, 0.86]; *p* = 0.004; I^2^ = 7 %). Subgroup analyses suggested the reduction of hepatic fibrosis to be more pronounced after ≤ 6 cycles of bevacizumab (0.60 [0.38, 0.94]; *p* = 0.03; I^2^ = 0 %) compared to > 6 cycles of bevacizumab (0.70 [0.30, 1.63]; *p* = 0.41; I^2^ = 36 %) and in case a high proportion of patients received an oxaliplatin-based chemotherapy regimen (0.52 [0.37, 0.75]; *p* < 0.001; I^2^ = 0 %) compared to a lower fraction of patients with an oxaliplatin-based chemotherapy regimen (0.70 [0.30, 1.63]; *p* = 0.41; I^2^ = 36 %). In these analyses statistical heterogeneity was caused by the study by Wicherts et al. [33]. Exclusion of this study completely resolved statistical heterogeneity (0.43 [0.19, 1.0]; *p* = 0.05; I^2^ = 0 %) in the subgroups of patients with > 6 cycles of bevacizumab and a lower fraction of oxaliplatin-based chemotherapy regimens.

In total, seven studies with a sample size of 1116 patients provided results on hepatic steatosis after preoperative chemotherapy with and without bevacizumab [10, 20, 26, 28, 29, 31, 33]. Pooled analysis of the results from these studies indicated no impact of preoperative chemotherapy with bevacizumab on hepatic steatosis (0.96 [0.63, 1.45]; *p* = 0.83; I^2^ = 30 %). The lack of association between preoperative bevacizumab administration and hepatic steatosis was confirmed throughout the performed subgroup analyses.

In further analyses the effect of preoperative chemotherapy with and without bevacizumab on complete (R0) resection of liver metastases and complete pathologic response was evaluated. These analyses revealed no significant association between preoperative bevacizumab treatment and R0 (0.71 [0.32, 1.59]; *p* = 0.40; I^2^ = 58 %) and complete pathologic response (1.51 [0.83, 2.75]; *p* = 0.18; I^2^ = 9 %).

### Functional recovery and perioperative outcome

Perioperative outcomes are summarized in Table 3. Meta-analysis showed no statistically significant difference in perioperative morbidity between patients with and without preoperative bevacizumab treatment with low statistical heterogeneity (1.10 [0.88, 1.37]; *p* = 0.39; I^2^ = 10 %). However, subgroup analyses revealed increased perioperative complications in patients who received preoperative chemotherapy with bevacizumab for more than six cycles (1.51 [1.08, 2.13]; *p* = 0.02; I^2^ = 0 %). Neither the duration between the last dose of bevacizumab and the date of surgery nor the kind of cytotoxic therapy that was combined with bevacizumab had a significant impact on perioperative morbidity. Meta-analysis just failed to show a significant impact of preoperative bevacizumab treatment on severe complications (1.39 [0.96, 2.01]; *p* = 0.08; I^2^ = 0 %).

**Table 3:** Perioperative outcomes reported in studies on preoperative chemotherapy with and without bevacizumab for CLM

Reference	Group	Duration of surgery (min)	Estimated blood loss (ml)	Morbidity (%)	Severe morbidity (%)	Wound compl. (%)	Liver failure (%)	Bile leakage (%)	Thromboembolic events (%)	Hospital stay (d)	Mortality (%)
Aussilhou	BEV-			50			15	5			5
	BEV+			55			18	9			0
Constantinidou	BEV-		650 (100–2200)	54	13	12	13		0	10 (4–32)	2
	BEV+		540 (50–2000)	48	12	14	10		5	10 (4–57)	0
D’Angelica	BEV-	-	500 (200–5000)	38	9	6				7 (n.r.)	0
	BEV+	235 (85–500)	300 (0–1500)	41	6	19					0
Kesmodel	BEV-	134 (69–408)	200 (50–1750)	43		25	7	2			2
	BEV+	139 (67–675)	250 (50–1950)	49		28	1	4			1
Lubezky	BEV-			47	9	2	5	9	4		0
	BEV+			35	11	2	1	8	5		2
Mahfud	BEV-	270 (237–302)	658 (407–908)	40	18	9	20		9	13 (8–18)	4
	BEV+	248 (224–270)	523 (399–646)	56	31	11	7		7	15 (11–20)	0
Millet	BEV-	300 (175–645)	500 (50–1700)	34	10		12			8 (5–21)	0
	BEV+	300 (156–540)	500 (150–1100)	56	12		22			9 (6–22)	0
Pessaux	BEV-	277 (±55)	548 (±233)	19		0	0	0		13.7 (±4.2)	0
	BEV+	321 (±160)	983 (±1476)	29		14	0	0		12.7 (±6.3)	0
Reddy	BEV-		600 (350–800)	39	25	7	26		5	7 (6–9)	4
	BEV+		425 (263–600)	44	28	10	18		3	8 (7–10)	3
Rong	BEV-			29		1	4	7	1	10 (±50)	
	BEV+			26		6	1	3	0	9 (±35)	
Tamandl	BEV-			34	7					9 (5–47)	0
	BEV+			44	11					8 (4–77)	0
Van der Pool	BEV-			32	6			4		7 (3–23)	0
	BEV+			25	8			2		6 (3–54)	0
Wicherts	BEV-	341 (±110)		36	5	14	2	3	1	11.3 (±7.1)	0
	BEV+	414 (±146)		43	16	8	2	6	2	13.6 (±14.4)	0
Zorzi	BEV-			54	39		31				8
	BEV+			42	16		26				5

Values are presented as percentages. Continuous data are presented as median (range) or mean (standard deviation) based on the kind of data presented in the original publication. n.r., not reported

Due to the role of VEGF-A in physiological wound healing, the influence of chemotherapy with bevacizumab on wound healing complications was analyzed. Meta-analyses revealed a significant association of preoperative bevacizumab administration and postoperative wound healing complications with no statistical heterogeneity (1.81 [1.12, 2.91]; *p* = 0.02; I^2^ = 4 %). Subgroup analyses indicated a more pronounced impact on wound healing, if > 6 cycles of bevacizumab were administered preoperatively.

Further, the effect of preoperative bevacizumab administration on postoperative liver dysfunction was analyzed. This analysis revealed a trend towards a decreased incidence of posthepatectomy liver failure in patients who received chemotherapy together with bevacizumab preoperatively (0.61 [0.34, 1.07]; *p* = 0.08 I^2^ = 6 %). Subgroup analyses failed to demonstrate an influence of the number of bevacizumab cycles and the time interval until surgery on the development of posthepatectomy liver failure. The reduction of this complication appeared to be more pronounced in studies with higher proportion of patients with irinotecan-based chemotherapy (0.61 [0.28, 1.30]; *p* = 0.20; I^2^ = 29 %). Sensitivity analyses revealed statistical heterogeneity in this subgroup of being caused by the study by Millet et al. [10] Removal of this study completely resolved statistical heterogeneity and indicated a statistically significant reduction of liver failure in the bevacizumab group (0.44 [0.21, 0.89]; *p* = 0.02; I^2^ = 0 %). In total, 13 studies with a cumulative sample size of 1329 patients provided data on perioperative mortality [7, 10, 20–23, 25–27, 30, 31, 33, 34]. The pooled analysis of the results from these studies did not show a significant impact of chemotherapy combined with vs. without bevacizumab on perioperative mortality after liver resection (0.60 [0.20, 1.82]; *p* = 0.37; I^2^ = 0 %).

## Discussion

It has been demonstrated previously that the chemotherapeutic agent oxaliplatin increases the risk of SOS [35]. This study shows that preoperative administration of bevacizumab is associated with a strong reduction of total SOS incidence as well as the incidence of moderate and severe SOS. Remarkably, subgroup analyses revealed that the reduced incidence of SOS was more pronounced in case ≤ 6 cycles of chemotherapy with bevacizumab were administered suggesting that the protective effect tapers off with time. The mechanisms by which anti-VEGF therapy reduces the development of SOS remain incompletely understood. Besides biologic processes related to oxidative stress, remodeling of the extracellular matrix and the coagulation cascade, gene expression analyses have suggested angiogenic pathways to be involved in the pathogenesis of SOS [36, 37].

It is an interesting finding of the present study that preoperative treatment with bevacizumab significantly reduced hepatic fibrosis but had no impact on hepatic steatosis. This effect appeared to be more pronounced for a shorter period of preoperative chemotherapy and in case anti-VEGF therapy was given together with oxaliplatin-based chemotherapy. Previous studies have already demonstrated an anti-fibrotic activity of anti-angiogenic agents in the hepatic parenchyma [38, 39]. Using a rat liver fibrosis model Wang et al. showed that sorafenib, a multiple receptor tyrosine kinase inhibitor that among others targets the VEGF receptor family (VEGFR-2 and VEGFR-3) and platelet-derived growth factor receptor family (PDGFR-beta and Kit) [40], reduces intrahepatic fibrogenesis. The anti-fibrotic effect of sorafenib may be mediated by targeting PDGFR which have been shown to play an important role in liver fibrogenesis [41]. Much less is known about the effects of the anti-VEGF antibody bevacizumab on remodeling of the extracellular matrix within the hepatic parenchyma. The results of the present study should therefore prompt further investigations elucidating the molecular mechnisms by which anti-VEGF therapy attenuated hepatic fibrosis in patients receiving systemic chemotherapy and, moreover, explore its potential as an anti-fibrotic agent in patients with liver fibrosis due to various etiologies.

Among other cytokines VEGF has been repeatedly shown to be involved in the process of liver regeneration [42, 43]. Introduction of anti-VEGF agents have therefore raised questions regarding the safety of bevacizumab in the peri-operative setting in patients undergoing liver resection due to potentially detrimental effects on the regenerative capacity of the liver. Preclinical studies indeed showed a slight impairment of liver regeneration by treatment with an anti-VEGFR2 antibody in a murine model of partial hepatectomy [44]. Interestingly, the results of the present study suggest the incidence of posthepatectomy liver failure of being less frequent in patients who receive chemotherapy together with bevacizumab. One must note that the above studies evaluated the role of VEGF and the effect of anti-angiogenic therapy on liver regeneration without concomitant use of cytotoxic chemotherapy. Furthermore, the decreased incidence of posthepatectomy liver failure after chemotherapy with bevacizumab may be mediated by protection from SOS and liver fibrosis.

Despite a beneficial effect of preoperative bevacizumab administration on parenchymal damage and functional recovery of the liver, we noted a significant increase in wound complications and total morbidity in case a high number of preoperative chemotherapy cycles was administered. Owing to these findings the positive effects of VEGF-targeted therapy on the hepatic parenchyma need to be weighed against potential risks. However, dissection of the molecular mechanisms responsible for decreased parenchymal damage might help to develop therapies specifically targeting pathways involved in chemotherapy-associated liver injury and in particular SOS.

Our study has several limitations. First, all studies included in the present meta-analysis were non-randomized studies and may therefore be affected by various sources of bias. Second, no uniform definitions as published before [45–47] were applied for the most relevant complications after hepatobiliary surgery. Third, we noted marked intra-and interstudy heterogeneity of the included studies with respect to the study designs, sample sizes, administered chemotherapy protocols and evaluated outcomes. We addressed these issues by robust methodology using a priori defined subgroup and sensitivity analyses together with a random effects model.

## Conclusion

In conclusion, the results of the present systematic review and meta-analysis confirmed the safety of chemotherapy together with the anti-VEGF antibody bevacizumab in the perioperative treatment of patients with CLM. The protective effects from SOS and hepatic fibrosis warrant further investigation and may at least in part serve as an explanation for the unexpected finding that treatment with bevacizumab reduces the incidence of posthepatectomy liver failure.

## Additional files

Additional file 1:
**Table S1.** Histological analysis and parenchymal damage reported in studies on preoperative chemotherapy with and without bevacizumab (DOC 55 kb) Additional file 2:
**Table S2.** Definition of fibrosis in available studies (DOC 43 kb)

## Abbreviations

CLM: colorectal liver metastases
PDGFR: platelet derived growth factor receptor
SOS: sinusoidal obstruction syndrome
VEGF: vascular endothelial growth factor
