# Supplementary material for: Impact of Bevacizumab on parenchymal damage and functional recovery of the liver in patients with colorectal liver metastases
Source: BMC Cancer. 2016 Feb 10;16:84. doi: 10.1186/s12885-016-2095-6 (PMC4750178; doi:10.1186/s12885-016-2095-6)
Supplement: Additional file 1: — Table S1. Histological analysis and parenchymal damage reported in studies on preoperative chemotherapy with and without bevacizumab (DOC 55 kb) [file 12885_2016_2095_MOESM1_ESM.doc]

**Additional file 1: Table S**1: Histological analysis and parenchymal damage reported in studies on preoperative chemotherapy with and without bevacizumab

| **Reference** | **Group** | **SOS [%]** | **Moderate/severe SOS [%]** | **Fibrosis [%]** | **Steatosis [%]** | **CR [%]** | **R0 [%]** |
| --- | --- | --- | --- | --- | --- | --- | --- |
| Aussilhou | BEV- |  | 30 | 20 | 0 |  |  |
|  | BEV+ |  | 55 | 0 | 10 |  |  |
| Constantinidou | BEV-  BEV+ |  |  |  |  | 12  12 | 28  38 |
| Klinger | BEV-  BEV+ | 52  42 | 35  10 | 17  10 |  | 4  10 | 100  93 |
| Millet | BEV-  BEV+ | 49  29 | 24  10 | 34  17 | 32  32 |  | 88  63 |
| Pessaux | BEV-  BEV+ | 24  14 | 19  10 | 19  14 | 24  0 |  |  |
| Reddy | BEV-  BEV+ |  |  |  |  |  | 86  92 |
| Ribero | BEV-  BEV+ | 54  27 | 28  8 |  |  | 12  11 |  |
| Rong | BEV-  BEV+ | 20  11 |  | 14  10 | 33  39 |  |  |
| Rubbia Brandt | BEV-  BEV+ | 77  64 | 62  31 | 52  31 | 40  39 |  |  |
| Van der Pool | BEV-  BEV+ | 64  49 | 28  8 | 40  35 | 25  12 |  |  |
| Vera | BEV-  BEV+ |  |  |  |  | 27  49 |  |
| Wicherts | BEV-  BEV+ |  |  | 45  53 | 6  7 | 8  3 | 53  42 |

SOS, sinusoidal obstruction syndrome; CR, Complete pathological response; R0, resection with negative margins

Values are presented as percentages.
