# Supplementary material for: Impact of Bevacizumab on parenchymal damage and functional recovery of the liver in patients with colorectal liver metastases
Source: BMC Cancer. 2016 Feb 10;16:84. doi: 10.1186/s12885-016-2095-6 (PMC4750178; doi:10.1186/s12885-016-2095-6)
Supplement: Additional file 2: — Table S2. Definition of fibrosis in available studies (DOC 43 kb) [file 12885_2016_2095_MOESM2_ESM.doc]

**Additional file 2: Table S2: Definition of fibrosis in available studies**

| **Reference** | **Fibrosis** |
| --- | --- |
| Aussilhou | > F2 |
| Klinger | > Grade 2 |
| Millet | Centrilobular/venular fibrosis |
| Pessaux | Perisinusoidal and portal |
| Rubbia Brandt | Centrilobular ⁄ venular fibrosis |
| Van der Pool | Perivenular fibrosis > 50% |
| Wicherts | Portal or portoportal |
